# New York Homœopathic Union

**Published:** 1897-02

**Authors:** Edmund Carleton

**Affiliations:** 62 West Forty-ninth Street, New York


### NEW YORK HOMŒOPATHIC UNION.

Editor Homœopathic Physician :—Owing to the illness of our Secretary it will be impossible to furnish a complete report of the regular monthly meeting of the New York Homœopathic Union, which was held on the 21st instant. One hour was devoted to consideration of Hahnemann's *Chronic Diseases* (pp. 184 *et seq.* Hempel).

In contrast with the old method of treating mixed psora and syphilis with Guaiacum followed by Sarsaparilla, followed by Mezereum, Dr. Finch spoke of the sequence often seen in homœopathic practice, namely: Sulph., Calc., Lyc., or Sulph., Sarsap., Sepia. Many cases then require a dose of Mercury.

Dr. Young—Swan's degree of strength marked M (of any remedy), does not act after Bœricke & Tafel's 200th centesimal has been exhausted.

Dr. Powel—After a higher potency of one maker's scale has ceased to act, a lower potency of another maker's scale may act.

According to recent custom the next hour was spent upon clinical reports and discussions. Dr. Vondergoltz being prepared with manuscript, read the following cases:

Case No. 479.—Miss J. G., twenty-six years old. Menses with thirteen and a half years; regular for three days up to sixteenth year. Since then patient is suffering.

August 22d, 1896.—Metrorrhagia for ten years. Blood clotted; when lessened thin, copious leucorrhœa, Thuja; metrorrhagia was stopped till August 26th. Sac-lact.

September 12th.—Patient has a black, fœtid hemorrhage every second day; felt miserably; unnatural hunger. Phosphorus.

September 24th.—Feels as if everything would fall out; bleeding. Sec-cor.

October 3d.—Patient feels well. S-l.

October 10th.—Only one show of blood; feels well. S-l.

October 17th.—Some drops of blood on the diaper. China.

October 24th.—Piercing pains in the left ovarian region; stitching down the leg; very depressed. Chamomilla.

October 31st.—Nausea. Ipec.

November 7th.—Sabina symptoms.

November 13th.—Much better. Sac-lact.

December 4th.—Crocus symptoms.

January 7th, 1897.—Patient was perfectly free from metrorrhagia for the whole month; no pain; feels cured. Patient ordered to return to office as soon as a single drop of blood will be noticed. (January 21st, 1897.)

[Patient told that she was treated and operated for her continuous metrorrhagia by Dr. Hanks at the Women's Hospital, where she stayed for nine weeks; was further operated by Dr. Florien Krug, at the German Hospital, and further was operated twice at Mount Sinai by Dr. Brettschaur, and then treated privately for three months by Dr. Brettschaur, etc.]

As I made the examination I was decided, if my treatment (as fully expected by me) would fail, to perform the vaginal hysterectomy as last resort.

*The Physical Examination revealed the following:* Uterus in retroflexion, small; cavity four inches deep, movable; endometrium soft, seems to be granular. The endometrium bleeds from the least touch. Cervix is patulous, open; so that with a reflector I can see far up to the flexion.

*Re-examination,* September 24th.—Uterus in retroflexion, os cervicis closed, consistency of organ harder, endometrium harder.

*Re-examination,* October 24th.—Uterus is easily brought in anteflexion in knee-chest posture, by air pressure from open vagina, uterine cavity three inches deep.

*Re-examination,* January 7th, 1897.—Uterus in anteflexion, $2\frac{4}{5}$ inches deep, not sensitive to touch, perametria free.

January 7th.—Patient discharged cured, so far from hemorrhage of the uterus for ten years. For the longest time free from hemorrhage all the time was eight days.

*Physical Examination of the whole organismus:* Patient is built in a strong bone frame; muscles are weak and anæmic; skin of the whole body looks yellow. The type of the whole appearance is masculine, especially the face and chest. The breasts less developed than in any male; arms and legs are comparatively well-developed; heart, lungs, and all other organs in good order.

*Obstetrical Record*, CASE NO. 476.—Mrs. St., forty-six years old, fourteenth pregnancy expected August 14th to 18th, 1896. I was called August 15th, 5 P. M., on account of beginning bleeding. As I examined patient I found cervix closed, but soft; fœtus in transverse position. As patient was in bad temper, anguish, restless, etc., I gave Chamomilla$^{30}$. As I called at 8 P. M., patient was quiet, bleeding was stopped, no labor-pains.

Eleven P. M.—I was hurriedly called, a hemorrhage had suddenly begun. As I came, patient was nearly exhausted; cold, moist face, etc. I sent immediately for Dr. S. to give Chloroform, as I saw that the woman must be delivered immediately.

As soon as Dr. S. came and the patient was under the narcosis, she was put in transverse position for version of the fœtus with extraction. After having cleaned and sterilized the vagina as quickly as possible, I examined preparatory. The cervix, still closed, was gradually dilated. I forced my hand up, and found as cause of the arterial bleeding the insertion of the placenta in the inner os cervicis. The velamina of the fœtus were unruptured; as I ruptured these a gush of water and blood burst forth. I grabbed now for the feet, and after having both well down and versed the fœtus, I extracted the child. I then loosened the placenta, and removed it, together with the water-bag. As the bleeding did not stop, I examined with hand and curette, and found that the outer layer of the water-bag was in toto retained. After having loosened it I was able to produce this velamen as an entirety, the uterine cavity fitting cast. Immediately the hemorrhage stopped. The weak and anæmic patient was now dressed and cleaned, and so finally got the desired rest. I left her with one dose Arnica at 3 A. M.

August 16th, 9 A. M.—Patient comparatively well.

August 17th—Patient shows symptoms of loss of blood—China.

August 18th to 20th—Patient improves gradually.

August 21st—Patient feels really well for the first time.

August 22d—Patient got up for the first time.

August 29th—Well. Discharged.

Dr. Clark had a case of menorrhagia, which he treated with a number of remedies, prescribing according to the character of the flow. At length he found these constitutional symptoms: cold flesh, but patient could not bear to be covered. Secale$^{200}$ in water, every hour, soon stopped the blood, but nausea ensued. Next month the same. He then gave a dose of Secale$^{1000}$, and all symptoms were soon cured.

Dr. Baylies mentioned a case of miscarriage, having a black, stringy discharge. *Crocus*$^{40m}$ ameliorated but did not cure. The same with a higher degree of strength. The patient had damp, cold feet, and could sleep better with them out of bed. Sulphur did not relieve. Neither did Secale. Then Crocus$^{cm}$ ameliorated. Physical examination had been refused. This the doctor now insisted upon (at the end of thirty-one days). Detritus was removed mechanically. Three days later, a flow of bright blood came on. At the same time he noticed fidgetty feet. A dose of Zinc cured all symptoms.

Dr. Young thought that the homœopathic physician should rely upon carefully applied materia medica.

Drs. Clark, Finch, Powel, Carleton, and others joined in the discussion. It then being ten o'clock, Dr. Vondergoltz was called upon to close the debate, in the course of which he remarked that there is nothing new under the sun. An old German book has recently come to light, in which the position now accorded to Trendelenberg is recommended.

Other clinical reports were necessarily postponed to next meeting.

Adjourned.

Yours truly,

EDMUND CARLETON.

62 West Forty-ninth Street, New York,
January 23d, 1897.

